# A coherent structural picture of the interaction of Tau with tubulin provides a link to its aggregation

**DOI:** 10.1016/j.jbc.2026.113086

**Published:** 2026-04-27

**Authors:** Benoît Gigant, Liza Ammar Khodja, Valérie Campanacci, Guy Lippens

**Affiliations:** 1 Université Paris-Saclay, CEA, CNRS, Institute for Integrative Biology of the Cell (I2BC), Gif-sur-Yvette, France; 2 TBI, Université de Toulouse, CNRS, INRAE, INSA, Toulouse, France

**Keywords:** Alzheimer's disease, amyloid, microtubule, microtubule dynamics regulation, structural model, tauopathy, Tau protein (Tau), tubulin

## Abstract

Tauopathies are a group of neurodegenerative diseases characterized by the presence of insoluble filaments of the Tau protein in the brain. In physiological conditions, Tau is involved in the regulation of microtubule dynamics. The study of its interaction with different tubulin assemblies, using various experimental approaches, leads to a seemingly disparate picture. Here, we propose to integrate this information into a model of how Tau participates in microtubule assembly and stabilization. Related to its intrinsically disordered nature, the binding of Tau to microtubules involves both specific interactions, along protofilaments, and nonspecific ones, with the C-terminal region of tubulin subunits. In addition, the recent determination of a Tau:tubulin structure provides a model for a functional dimer of Tau targeting a microtubule aperture between protofilaments. Therefore, Tau regulates microtubule dynamics by modulating both longitudinal and lateral contacts. Finally, we discuss a possible connection of this dimer of Tau with its oligomerization, whether physiological or pathological.

The presence in the brain of fibrillary tangles made of the Tau protein is a molecular feature of Alzheimer’s disease (1, 2). The principal component of these tangles consists of paired-helical filaments (PHFs) while less abundant straight filaments are also found (3, 4). More generally, Tau aggregates are a hallmark of an ensemble of neurodegenerative disorders known as tauopathies (1, 2). In these aggregates, Tau adopts disease-specific molecular conformers, which have led to a structure-based classification of tauopathies (5, 6). Although mostly studied in the framework of these diseases, Tau was nevertheless first discovered as a “Tubulin associated unit” that copurifies with the αβ-tubulin heterodimer (tubulin) and regulates microtubule (MT) dynamics (7). Although cryo-EM has been the method of choice for the characterization of the disease-associated *ex vivo* filaments (8, 9), structural information on the role of Tau in MT assembly has not come only from cryo-EM (10) but also from other approaches including liquid and solid-state NMR spectroscopy (11, 12, 13, 14, 15, 16), electron paramagnetic resonance (17), fluorescence-based methods (18), and more recently X-ray crystallography (19). One important issue is that Tau’s interaction with a single tubulin molecule, with small tubulin assemblies, or with MTs, either preformed or following coassembly, all need to be considered to enhance our understanding of its mechanism. Here, we propose to bring together this ensemble of information into a coherent picture of how Tau participates in the essential cellular process that is MT assembly and stabilization. We also propose a possible connection with tauopathy-associated oligomerization.

Although there is a single *MT-associated protein Tau* (*MAPT*) gene, alternative mRNA splicing leads to several isoforms of Tau. The predominant isoforms comprise up to 441 residues (Tau441; Fig. 1). However, less abundant higher molecular weight isoforms, known as big Tau (20), also exist and have been recently shown to be involved in neuronal dysfunction under pathological conditions (21). The C-terminal “assembly domain” of Tau comprises the regions involved in the interaction with the MT. It includes four imperfect repetitions of a 31- to 32-residue–long motif, known as the MT-binding repeats (or MTBRs). The repeats are usually named R1 to R4, with R2 being coded by an alternative exon, hence absent in so-called 3R isoforms and present in 4R ones. Originally, they were subdivided into a semiconserved 18-residue long C-terminal region, and an N-terminal “inter-repeat” stretch (22). The inter-repeat stretch appears to be more divergent in R1. In R3, it comprises the ^306^VQIVYK^311^ hexapeptide (numbering is according to Tau441), termed PHF6 because of its contribution in initiating PHF assembly (23), whereas a related ^275^VQIINK^280^ PHF6∗ motif is found in R2 (24) (Fig. 1). Despite the name, constructs limited to all or part of the MTBRs bind rather weakly to taxol-stabilized MTs and “flanking” segments of a proline-rich region (PRR), N-terminal to the MTBRs, and/or of the C-terminal downstream region are needed for high affinity binding to these prestabilized MTs (25, 26, 27, 28). Because it is weakly homologous to the repeats, the segment following R4 is sometimes referred to as R’ (26) (Fig. 1).

**Figure 1:**
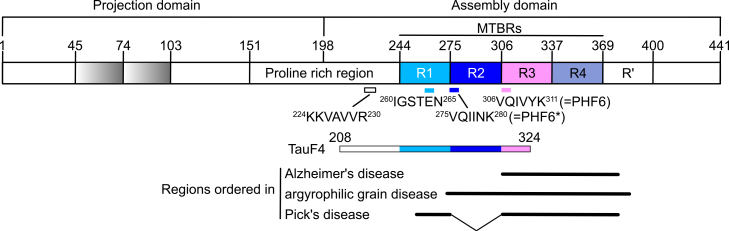
**Domain organization of Tau 441, the 441-residue–long isoform of human Tau.** Shorter variants result from the splicing of exons coding for one or two inserts in the N-terminal projection domain (*gray*) or for the second repeat in the MTBR region (R2, *dark blue*). The position and sequence of Tau motifs discussed in the article are indicated. The TauF4 construct, from Ser208 to Ser324, is also illustrated. The regions ordered in disease-associated filaments are indicated in the cases of Alzheimer’s disease (4) (a 3R+ 4R tauopathy; PDB ID: 5O3L), of argyrophilic grain disease (5) (a 4R tauopathy; PDB ID: 7P6D), and of 3R-specific Pick’s disease (90) (PDB ID: 6GX5). MTBR, microtubule-binding repeat; Tau, tubulin-associated unit.

One defining characteristic of the Tau protein is its dynamic nature. Indeed, although the term was not coined at that time, Tau was early on pictured as an intrinsically disordered protein (29). Molecular flexibility not only concerns isolated Tau (30) but also Tau in assemblies, either within fibrils or bound to tubulin. For instance, in cryo-EM structures of Tau filaments, the regions outside the folded core remain disordered and form a so-called fuzzy coat (4). A method well suited to study dynamic assemblies is NMR spectroscopy, as discussed in the next section.

## The fuzzy interaction of the PPR with the C-terminal region of tubulin subunits

The binding of Tau to MTs involves both specific and dynamic interactions. In this section, we will focus on the PRR which, despite considerably contributing to the affinity, remains mobile in complexes with tubulin. In accordance with early biochemical characterization (25, 26, 27), the first NMR spectroscopy experiments using isotope-labeled Tau in the presence of taxol-stabilized MTs defined the interacting region as the MTBRs plus the C-terminal part of the PRR, taking as a threshold a remaining NMR signal less than 20% of that of the unbound state (11). Using assemblies far smaller than MTs, the picture could be refined to a residue-level resolution (14). These assemblies comprised one or two tubulin heterodimers which are bound to a stathmin-like domain (SLD) protein to prevent further tubulin self-association (31). They include in particular the curved protofilament-like T_2_R complex, formed by two tubulin molecules and one SLD of the RB3 protein.

When labeled Tau constructs are mixed with T_2_R in the NMR tube, many resonances of residues in the PRR remain visible but are shifted. One example is the ^224^KKVAVVR^230^ peptide (Fig. 1), which, when embedded in a larger construct, is an important contributor for Tau’s binding to MTs (27, 32). The resonances of these residues in a protein comprised of the 208 to 324 stretch of Tau (named TauF4, a construct derived from the result of a screen for the identification of Tau fragments interacting tightly with T_2_R and used as a simplified version of Tau) (28) (Fig. 1) could be explicitly assigned by triple-resonance NMR experiments (14). These require a minimal mobility for line narrowing, thereby arguing against the peptide adopting a defined structure in interaction with T_2_R. By comparison, the resonances of MTBR residues are more affected both in term of the proportion of disappearing resonances and of the broadening of the remaining signal (14, 28). This view is consistent with cryo-EM data on Tau bound to MTs, where the PRR is not visible in the resulting maps (10). It should be noted, however, that the Tau constructs used to derive atomic models in cryo-EM experiments comprised four identical copies of either the first or the second repeat inserted between stretches of 45 residues upstream and 33 residues downstream the MTBR region. Hence, these flanking segments are not expected to follow the same binding periodicity compared to the repeats and their signal might have been blurred during data processing.

**Figure 2:**
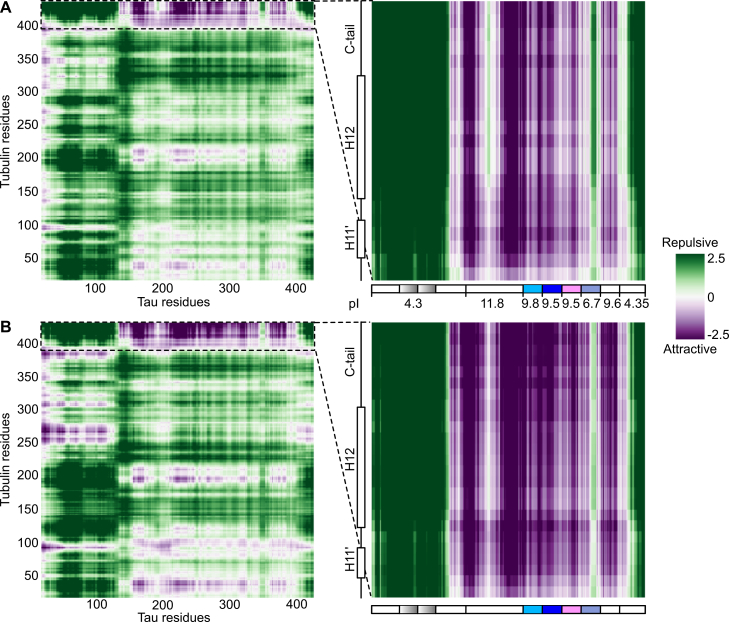
**Predicted intermolecular interaction maps (intermaps) of Tau 441 with tubulin subunits.***A*, intermaps with full-length α-tubulin (α1b isotype) (*left*) and with the α-tubulin C-terminal region (from residue 400, *i.e.*, a stretch comprising the H11′ and H12 helices and the disordered C-tail (97)) (*right*). In the *right panel*, the domain organization of Tau 441 taken from Figure 1 is indicated, together with the predicted pI of the different regions. *B*, intermaps with β-tubulin (β2b isotype) as in *panel A*. These intermaps were obtained with the online version of FINCHES (https://.finches-online.com/) using default settings (33). *Right*, FINCHES color code, from *dark green* (repulsive) to *dark violet* (attractive interactions). C-tail, C-terminal tail; FINCHES, first-principle interactions *via* chemical specificity; Tau, tubulin-associated unit.

Intermolecular interactions driven by disordered regions can now be analyzed using sequence-based prediction tools, as implemented in the “first-principle interactions *via* chemical specificity” program (33). These computational approaches aim to identify nonspecific, “chemical” interactions, where (at least) one partner remains disordered in the complex, compared to “site specific” interactions between two ordered entities. First-principle interactions *via* chemical specificity (33) predicts that the acidic C terminus of both α and β-tubulin subunits, comprising the H12 helix and the disordered C-terminal tail (C-tail), associates nonspecifically with Tau (Fig. 2), in agreement with the NMR study of the interaction of Tau with a C-terminal peptide of α1a-tubulin (13). Removing the C-tail of tubulin subunits by subtilisin proteolysis (34) consistently reduces the amount of Tau which coassembles with tubulin (35) and decreases the affinity of TauF4 for T_2_R by two orders of magnitude (14).

On the Tau side, two stretches of the PRR are likely to be involved in these nonspecific interactions (Fig. 2), a prediction supported by a recent study of the interaction of the PRR with tubulin using hydrogen-deuterium exchange coupled to mass spectrometry (36). In addition, the repeats R1 and R2, and to a lesser extent R3, along with the R′ might also contribute. This pseudo-repeat, beyond the MTBRs, together with the PRR would form the “jaws” positioning the repeats on the MT (12, 26, 37). The proposal that several Tau segments are engaged with the C-terminal region of tubulin is again consistent with the aforementioned NMR study (13), where the authors estimated that 1 Tau molecule can interact *in vitro* with up to eight tubulin polypeptides. By contrast, repulsive interactions are predicted between the C terminus of tubulin and the other regions of Tau, namely the N-terminal part upstream of the PRR, the R4 repeat, and the C terminus following the R’. It thus appears that the electrostatic character of Tau segments is the main driver of the nonspecific interactions with tubulin, with the positively charged regions predicted to interact, unlike those having an acidic pI (Fig. 2). This scheme agrees with the higher binding affinity of Tau fragments for MTs and their improved ability to promote tubulin assembly compared to Tau441 (28). This is particularly the case of TauF4 which is constituted of major regions involved in attractive interactions with tubulin (Fig. 2).

To summarize, both experimental data and computational approaches indicate that the PRR of Tau contributes to the binding to tubulin through nonspecific interactions with its acidic C-terminal region, including the disordered C-tails. Other regions of Tau, also predicted to bind dynamically, have instead been found to associate specifically with tubulin, as we will see in the following sections.

## How Tau promotes tubulin assembly

**Figure 3:**
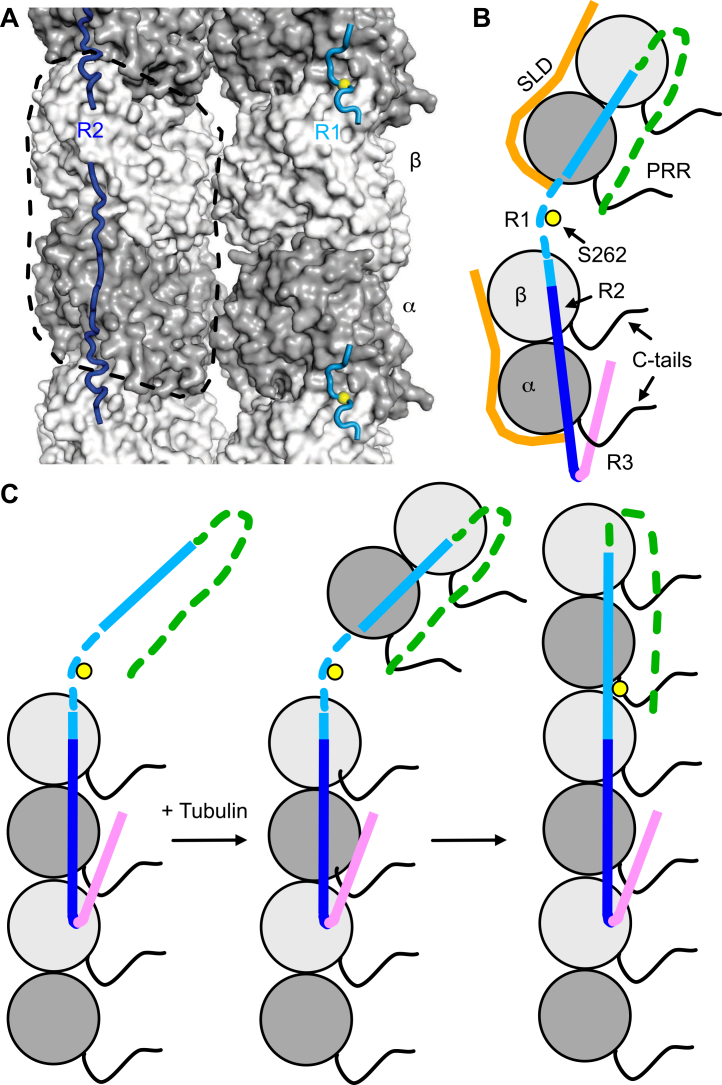
**Model for TauF4 promoting tubulin:tubulin longitudinal contacts leading to protofilament elongation.***A*, cryo-EM model of R1 and R2 bound to the MT (PDB ID: 6CVJ and 6CVN, respectively) (10). View from the outside of the MT and two protofilaments are shown, with R1 drawn on the protofilament on the *right*, R2 on that on the *left*. The α and β subunits are in *dark gray* and *light gray*, respectively. A tubulin heterodimer is delineated by a *dashed line*. The Cα of Ser262 in R1 is shown as a *yellow sphere*. Figures 3*A* and 5 were prepared with PyMOL (https://www.pymol.org). *B*, model of TauF4 interacting with two tubulin:SLD 1:1 complexes. The SLD is in *orange*, Tau repeats included in TauF4 are colored according to Figure 1 and the PRR is in *green*. R1 and R2 are positioned according to the cryo-EM models, the PRR is in dynamic interaction with the tubulin C-tails. R3 is tentatively positioned as interacting with the C-tails as well. *C*, TauF4 located at the plus end of a MT according to panel B (*left*, one protofilament of two tubulin molecules is drawn). R1 and the PRR are available to capture incoming tubulin heterodimers (*middle*), promoting MT elongation (*right*). C-tail, C-terminal tail; MT, microtubule; SLD, stathmin-like domain; Tau, tubulin-associated unit.

One of the best characterized properties of Tau, supported by an ensemble of structural approaches, is the stabilization of tubulin:tubulin longitudinal contacts. In this process, the Tau repeats play the key role. In the previous section, we emphasized the dynamic interaction of the PRR with tubulin. By contrast, in NMR experiments, residues in the N-terminal section of the MTBRs, and in R1 in particular, dropped below detection when mixing labeled TauF4 with T_2_R (14), indicating an immobilization that points to specific interactions with tubulin. These interactions have been visualized by cryo-EM associated with molecular modeling (10), with R1 in an extended conformation bound along protofilaments, at the exterior of peloruside-stabilized MTs (Fig. 3*A*). A similar but more complete cryo-EM model has been obtained for the second repeat with a dedicated construct containing four copies of R2. This repeat binds to one α-tubulin and the two adjacent β subunits, leading to an overall stoichiometry of one repeat per tubulin heterodimer (Fig. 3*A*).

The NMR picture changes when using assemblies comprising a single tubulin molecule as binding partners of TauF4. In this case, while resonance signals for residues in the PRR do not change much and those in R3 were found to be even weaker, those for R1 residues centered on the ^260^IGSTEN^265^ segment (Fig. 1) are now detected (14). A role of this motif was previously inferred from experiments showing that short peptides encompassing the ^260^IGSTEN^265^ residues stimulate MT assembly, although with a low efficiency (38, 39, 40). These NMR results initially led us to propose a model according to which TauF4 interacts first with a single tubulin. A turn centered on the first repeat would then serve as a hook to anchor incoming tubulin molecules to an elongating MT. In agreement with the cryo-EM model of Tau decorating the MT (10), TauF4 finally stretches out along the protofilament and thereby stabilizes longitudinal contacts (9, 14). The strengthening of these interactions is also supported by experiments showing that Tau favors the formation of tubulin rings (41), enhances the GTPase activity of colchicine-bound tubulin (14), and stabilizes intermediates in MT assembly and disassembly (42).

The availability of cryo-EM models for the interaction of R1 and R2 with the MT (10) (Fig. 3*A*) but also diffusion data obtained by fluorescence correlation spectroscopy (43, 44) suggest an alternative model. The hypothesis relies on one TauF4 molecule interacting with two complexes comprising one tubulin bound to an SLD (Fig. 3*B*), the tubulin molecules being unable to establish longitudinal contacts because of the N-terminal peptide of the SLD partner capping the α-tubulin surface involved in these interactions. Hence the ^260^IGSTEN^265^ segment, which targets an inter-tubulin region along protofilaments when bound to the MT would remain disordered and hence NMR visible. In the condition of MT assembly (*i.e.*, in the absence of a tubulin-sequestering protein), TauF4 would occasionally target the MT plus end, engaging its R2 and R3 repeats, whereas the PRR and R1 would be available to capture incoming tubulin heterodimers to elongate the protofilament (Fig. 3*C*). A solid-state NMR study also identified the PRR as the most mobile segment of the MT-interacting regions of Tau (15) but suggested a progressive immobilization from the PRR to R′, whereby the latter pseudo-repeat was modeled in the low-resolution cryo-EM map of full-length Tau on the MT. This study would extend the region targeting free tubulin for MT elongation from the PRR to R4. Whatever the region bringing in novel tubulin molecules through a “fly-casting mechanism” (45), this general model is reminiscent of that proposed for the XMAP215 family of proteins. These MT polymerases contain several TOG domains, which are specific either to free tubulin or to tubulin within MTs. The latter anchor XMAP215 to the protofilament’s end, while the former bind tubulin molecules to be incorporated into the growing MT (46).

Interestingly, Ser262 which belongs to the ^260^IGSTEN^265^ motif within R1 can be phosphorylated by the MARK kinase, leading to a decreased affinity for MTs (47). In the cryo-EM model, Ser262 interacts with Glu434 of α-tubulin, providing a basis for a destabilizing effect of Ser262 phosphorylation (10). It is equally possible that the ability to “fish” an incoming tubulin molecule is altered following this posttranslational modification.

Taken together, structural and biochemical results indicate that the Tau repeats favor longitudinal contacts within protofilaments, a feature contributing to the role of Tau in MT assembly. The hypotheses we have summarized here for Tau as a MT polymerase imply its targeting to the plus end of the MT and its ability to fish soluble tubulin heterodimers. The concentration of tubulin in the cells is in the range of 20 to 25 μM (48, 49), of which 5 to 15 μM are in soluble form (48, 50, 51). The concentration of Tau in neurons has been estimated to be 2- to 10-fold lower than that of tubulin (15, 52). In addition, estimates of the Tau:tubulin dissociation constant range from nanomolar to micromolar (14, 18, 53). All these features are consistent with the formation of Tau:tubulin complexes in physiological conditions. However, Tau binds more tightly to MTs than to soluble tubulin (28). Moreover, it preferentially targets the MT lattice rather than its ends (25, 54, 55). It is therefore possible that this fly-casting mechanism mostly concerns short MTs just after their nucleation, a step also stimulated by Tau (56). This scheme lines up with *in vitro* experiments, where Tau has been shown to stabilize MTs and to decrease the catastrophe frequency, with a lesser effect on the direct promotion of MT assembly (57, 58).

## The PHF6 region as a modulator of MT lateral contacts

**Figure 4:**
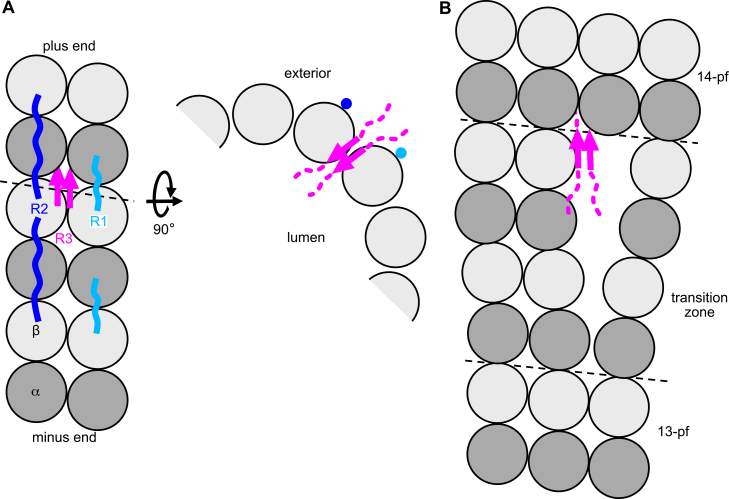
**How Tau stabilizes MTs**. *A*, two binding modes of Tau repeats to the MT. *Left*: lateral view from the outside of the MT. A stretch of two protofilaments of three tubulin heterodimers is shown. *Right*: view along the MT axis. Tau stabilizes longitudinal contacts by binding along protofilaments as shown by cryo-EM for the R1 and R2 repeats (10). The model for the R3 PHF6 targeting an aperture between protofilaments and stabilizing lateral contacts derives from the crystal structure of tubulin-bound DARPin-R3 chimera (19). It implies that the N-terminal and C-terminal regions of Tau (*dashed* in the *right panel*) would be outside and inside the MT, respectively. The repeats are colored according to Figure 1. *B*, model for Tau binding at a lattice defect due to a change in the number of protofilaments. DARPin, designed ankyrin repeat protein; MTBR, microtubule binding repeat; Tau, tubulin-associated unit.

In addition to the well-established binding of Tau to the MT outer surface along protofilaments (Figs. 3*A* and 4*A*) (10, 59, 60), a complementary model according to which Tau would interfere with lateral contacts within MTs has recently emerged from structural data (19). Indeed, different modes of interaction have been reported. In particular, Tau has been proposed to bind across protofilaments (36, 61, 62, 63) and to the MT lumen (61, 64). Competition experiments with vinblastine binding raised the hypothesis that Tau targets the inter-tubulin interface within protofilaments as well (16). Different approaches further indicate that Tau has more than one binding mode depending on the tubulin target. For instance, a mutation such as P332L in the R3 repeat enhances the interaction with soluble tubulin up to 25-fold but has hardly any influence on the binding strength to preformed MTs (18), suggesting that interacting surfaces of soluble tubulin are hidden upon MT assembly. Moreover, when coassembled with tubulin, Tau partitions between populations of molecules which dissociate rapidly from MTs and of those which bind virtually irreversibly (65). Consistently, about half of Tau molecules diffuse on MTs, whereas the others are static (66). In addition, Tau decorates MTs in a nonuniform way and can form, according to different studies, small oligomers of 2 to 3 molecules (67), medium-size patches comprising 3 to 20 molecules (68), or unsaturated clusters (69) forming envelopes around MTs (70, 71, 72).

The crystal structure of a Tau fragment bound to tubulin recently allowed us to identify a new binding site (19). These data have been obtained with a fusion protein linking a tubulin-specific designed ankyrin repeat protein (73), aiming to keep the complex with tubulin monodisperse (31), to a Tau R3 peptide comprising the PHF6 motif. Assuming that the interaction of R3 with tubulin should resemble that of the R1 and R2 repeats with MTs, the design was guided by the corresponding cryo-EM models (10). It turned out that, in the crystal, the Tau fragment binds “laterally” to tubulin. Strikingly, such a binding was not observed with R1-, R2- or R4-based constructs, suggesting a specificity for R3 (19). When transposed to the MT, this binding corresponds to the targeting of an aperture between protofilaments, at the corner of four tubulin heterodimers (Fig. 4*A*). Remarkably, two R3 PHF6 peptides bind simultaneously as two parallel β-strands, reminiscent of the first intermediate amyloid (FIA) of Tau filaments assembled *in vitro* (74) and of fibrils from dopaminated Tau which display the smallest structured core observed to date (75) (see below). Interestingly, NMR spectroscopy studies of TauF4 in the presence of T_2_R indicated that the PHF6 transiently targets different locations as if it were scanning the tubulin surface but without finding a high affinity binding site in this assembly devoid of lateral interactions (14).

Does the Tau:Tau binding in a MT pore enhance the interactions between both protofilaments that carry 1 Tau molecule? If so, this would represent an attractive mechanism to stabilize MTs (76, 77) because these contacts are weaker than longitudinal ones and are lost first during MT shortening (78). Tau has indeed been proposed to strengthen such lateral contacts (36, 42, 61, 62, 63), based in particular on the observation that it stabilizes tubulin sheets (62) and, in addition to promoting the formation of tubulin rings (41), also favors their stacking (63), both assemblies being highly dependent on lateral interactions. These studies mostly proposed that a single Tau molecule connects adjacent protofilaments. A structural model of this mechanism was however missing. Our results (19) provide such a model and suggest that two PHF6 motifs cooperate to reinforce lateral contacts, and hence protect MTs from disassembly, as observed (37, 79, 80).

The model of the PHF6 peptides pointing from the outside toward the lumen of the MT implies that part of Tau has to be inside the MT (Fig. 4*A*). Although it has been shown that proteins can access the MT lumen after assembly (81), such a binding is more easily conceivable if it occurs during assembly. It could therefore be enriched in more dynamic regions of the MT, the alternation of periods of growth and shortening enhancing the probability for Tau to be incorporated. In agreement with this, Tau and MAP6 compete for MT binding in neurons, with MAP6 found predominantly in the proximal region of the axon, characterized by more stable MTs, and Tau enriched in the distal part, where more labile MTs are found (82, 83). There, Tau has been shown to favor MT growth. Indeed, in Tau-depleted neurons, fewer end-binding protein comets, which mark growing MT ends, are observed (82). However, the exact molecular function of Tau in the axon remains to be clarified. Does it make MTs more dynamic? Or does it stabilize to some extent their labile regions, which would be even more labile in the absence of Tau?

An alternative way to access MT pore and lumen is through lattice defects (84). Interestingly, Tau generates such defects by favoring the transition to MTs having 14 protofilaments from the more common 13-protofilament MTs (79) (Fig. 4*B*). Stabilizing lattice defects or facilitating their repair could contribute to Tau function since such mechanisms are efficient ways to protect MTs from disassembly (85).

## Tau molecules trapped in the MT lattice: implications for Tau oligomerization

The model of two Tau molecules binding between protofilaments (19) (Fig. 4) is also useful in the context of the physiological oligomerization of Tau. Indeed, Tau forms patches on MTs, which could be as small as dimers to as big as unsaturated clusters (67, 68, 69, 70, 71). Although these studies imply a continuum of sizes, the connection between small and large assemblies is not clear. The model (Fig. 4) directly provides a structural rationale for the formation of the smallest oligomers (dimers), which have been visualized in cells under physiological conditions (67) and which also form *in vitro* in a MT-dependent manner even at low Tau:tubulin molar ratios (86). It suggests a scenario whereby these two Tau molecules, anchored in the MT, constitute a nucleus from which larger oligomers will grow, likely through Tau:Tau interactions (86, 87) and possibly involving the Tau N-terminal region (70, 88). This scenario would also give an explanation why the Tau envelopes reform at the same locations on the MT after their dissolution by 1,6-hexanediol (71). Indeed, binding across protofilaments, which likely occurs as Tau coassembles with tubulin or in a repair event as mentioned above, would in addition emulate an irreversible association unless MTs disassemble. This model therefore also agrees with Tau molecules that cannot be displaced from MTs, a population only observed in coassembly experiments but not when Tau is added to preformed MTs (65).

## Tau molecules trapped in the MT lattice: a link with Tau aggregation?

It has been hypothesized that tauopathy-associated filaments could be initiated on MTs (67, 69, 89). In this scenario, could the Tau dimer trapped in the MT pore constitute a “functional minimal amyloid” in physiological conditions that would become a nucleus for the formation of such aggregates in pathological conditions? In this section, we will consider how the model of a Tau dimer targeting a MT hole can shed light on this issue.

Strikingly, the ordered region in the filaments from Alzheimer’s disease brain patients starts at the beginning of the PHF6 (4), as does the ordered part of the R3 stretches in the structure of tubulin bound to the R3 chimera (19). In the case of Alzheimer’s disease, this explains why 3R and 4R isoforms, although different in the segment N-terminal to the R3 repeat, are both incorporated in the filaments (4). By contrast, in isoform-specific filaments, the region upstream to R3 can also be ordered. This is the case of R1 in filaments from Pick’s disease (90), a 3R tauopathy, or of R2 in 4R tauopathies (5, 91) (Fig. 1).

**Figure 5:**
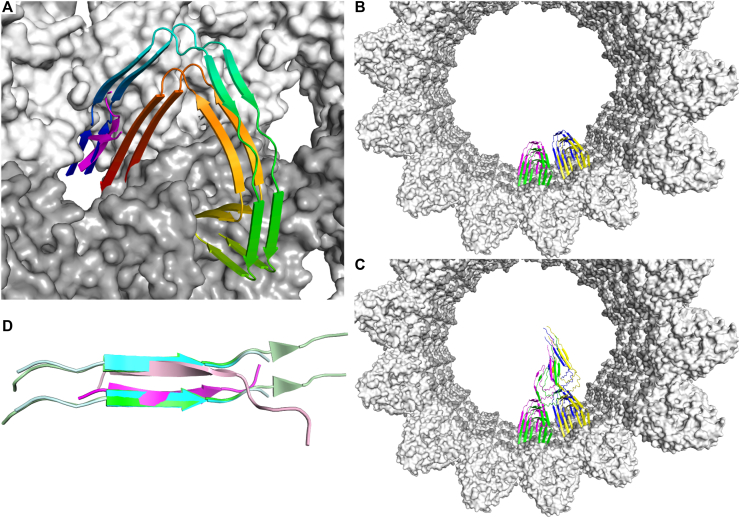
**A model of Alzheimer’s disease filaments anchored in the MT.***A*–*C*, Alzheimer’s disease filaments have been superimposed to R3 modeled between MT protofilaments. *A*, close-up view from the lumen of the MT (PDB ID: 7SJ7). The Tau filament (4) (PDB ID: 5O3L; two rungs of Tau molecules are shown) is rainbow-colored from *blue* (Val306-Lys311 β-strand) to *red* (Gly367-Phe378 β-strand). R3 from the tubulin-bound DARPin-R3 structure (19) (PDB ID: 9F07) is in *magenta*. *B*, Tau filaments modeled as in *panel A* are shown at four neighboring sites, each in a different color. *C*, same view as in *panel B*, but with one single Tau molecule of the second PHF filament also depicted. *D*, comparison of the R3 dimer bound to tubulin (the strand used for superposition is in *magenta*, the second one is in *pink*) with the first intermediate amyloid (FIA) (74) (PDB ID: 8PPO; *green*) and with dopaminated Tau fibrils (75) (PDB ID: 9MC2; *cyan*). In the case of the Tau filaments, two strands from one protofilament are drawn, with the PHF6 hexapeptides in brighter color. MT, microtubule; PHF, paired-helical filament; Tau, tubulin-associated unit.

Modeling the structured core of Alzheimer’s disease filaments in the MT, with the R3 PHF6 hexapeptide positioned between protofilaments as a guide, indicates that there is enough space in the MT lumen to accommodate the PHFs (Fig. 5, *A*–*C*). As expected, because of the Tau fold in these filaments, the C-terminal end of the Tau molecules anchored in the MT wall would also be located near the MT aperture, but pointing to the outside (Fig. 5*A*). Hence, only the region comprising the third and fourth repeats and the downstream R′ pseudo-repeat would be in the MT lumen, leading to a model with both the ∼300 N-terminal residues and the ∼50 C-terminal residues of the 441-residue–long isoform being outside the MT. Interestingly, these regions of Tau comprise many phosphorylation sites associated with Alzheimer’s disease (92, 93) and targeted in particular by the Gsk3β kinase (94).

Nucleation is the rate-limiting step in amyloid formation (95). In addition, in the case of Tau, a time-resolved cryo-EM study of its oligomerization has shown that the amyloid species go through several intermediates before maturing into Alzheimer-like filaments (74). The first characterized filament (called FIA, see above) appeared after 90 min and predominated after 2 h, but was no longer observed after 160 min incubation in this *in vitro* experiment using a construct comprised of residues 297 to 391 of Tau. The main secondary structural element present in this FIA is a β-sheet limited to the PHF6, reminiscent of what we have observed in the structure of R3-based Tau construct bound to tubulin (19). Strikingly, this minimal core has also been observed in fibers from dopaminated Tau (75) (Fig. 5*D*). All these data point to the PHF6 dimerization as the first event of the polymerization of Tau. The PHF6 dimer in the crystal adopts a conformation that is however not exactly that of the PHF6 in the FIA. It could represent a species which precedes the FIA, but which would be too sparsely populated, too transient or too small to be captured by cryo-EM. Taken together, the MT, by trapping two Tau molecules in an aperture between protofilaments, facilitates their inter-molecular interactions that could lead to filament nuclei; hence it lowers the kinetic barrier for the initiation of Tau aggregation. Of course, the elongation of such precursors would be possible only with their release, which is compatible with the decay of the neuronal MT cytoskeleton preceding the onset of other ageing hallmarks (96). These hypotheses should form the basis of future studies.

## Conclusion

In our previous review on “elucidating Tau function and dysfunction in the era of cryo-EM,” we mainly focused on the tsunami of novel *ex vivo* structures of disease-specific Tau fibrils (9). Structural information on Tau:tubulin complexes was already known, but the prevailing view was that Tau would dissociate from the MT, possibly as a consequence of ill-defined phosphorylation or other posttranslational modification events, before proceeding to aggregation. Here, we rather focus on the details of the interaction of Tau with tubulin, stressing that “tubulin” stands for different oligomeric forms that this protein adopts (a single heterodimer, two-tubulin complexes, larger oligomers, protofilaments, or assembled MTs) and that Tau has to adapt to these different targets. It should be noted that, at present, we mainly have a structural view of Tau fragments, often comprising part of the PRR and/or the MTBR region, in interaction with defined tubulin assemblies. The challenge will be to study the interaction of full-length Tau with various tubulin species along the MT assembly pathway.

The Tau:tubulin interaction is characterized by three general features. First, the fuzzy character of the interaction seems a recurrent theme, and applies first to the PRR interacting with the C-tails of tubulin subunits. Advances in sequence-based prediction of intermolecular interactions driven by disordered regions can now capture this part of the interaction (33). Second, cryo-EM together with molecular modeling has elucidated the more ordered interaction of repeats at the exterior of the MT (10), and although challenged by solid-state NMR, notably on the relative mobility of the different repeats when in interaction with a stabilized MT (15), has identified the crucial ^260^IGSTEN^265^ motif at the interface of two tubulin molecules. Solution state NMR of the TauF4 construct with capped tubulin assemblies agrees with this picture, whereby TauF4 would capture two tubulin heterodimers and bring them together with the ^260^IGSTEN^265^ segment at their interface (14) (Fig. 3). Although contributing to the nascent MT, Tau is not generally considered as a MT polymerase. Therefore, the interactions mediated by its ^260^IGSTEN^265^ motif are expected to stabilize longitudinal contacts in the MT core rather than directly favoring assembly. Finally, the recent crystal structure of an R3 fragment bound to tubulin in such a manner that, when transposed to the MT, two PHF6 peptides would dimerize at the lateral interface of adjacent protofilaments provides an additional view of Tau as a factor that stabilizes MTs (19). The most intriguing hypothesis is that this PHF6 dimer could serve as a nucleus for Tau assembly, thus unifying both the function and dysfunction of this remarkable protein.

## Conflict of interest

The authors declare that they have no conflicts of interest with the contents of this article.
